# Supplementary material for: Network Analyses of Brain Tumor Patients’ Multiomic Data Reveals Pharmacological Opportunities to Alter Cell State Transitions
Source: bioRxiv. 2024 May 10:2024.05.08.593202. Preprint. [Version 1] doi: 10.1101/2024.05.08.593202 (PMC11100715; doi:10.1101/2024.05.08.593202)
Supplement: Supplement 1 [file media-1.pdf]

Network Analyses of Brain Tumor Patients' Multiomic Data Reveals Pharmacological Opportunities to Alter Cell State Transitions

SUPPLEMENTARY INFORMATION

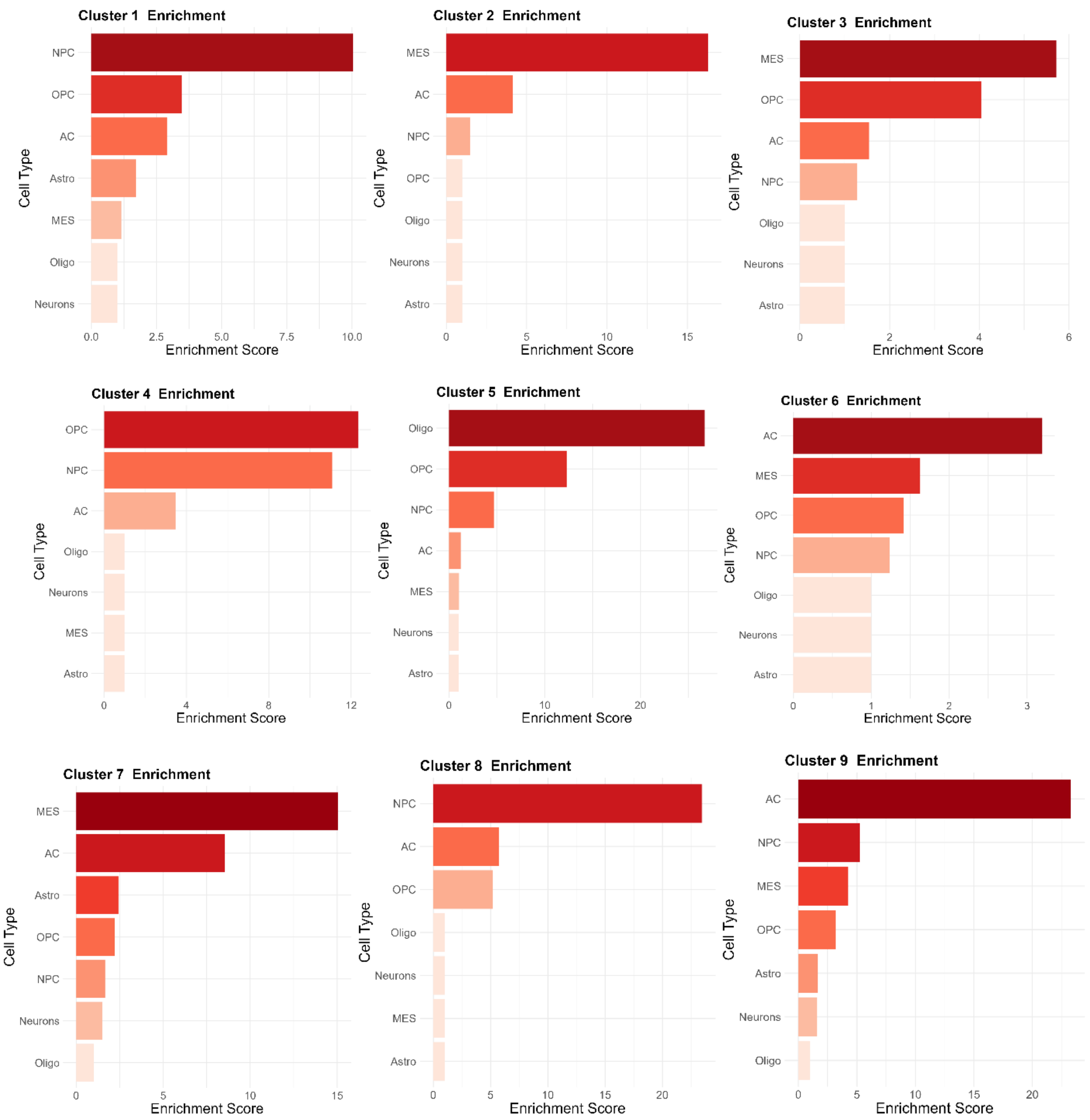

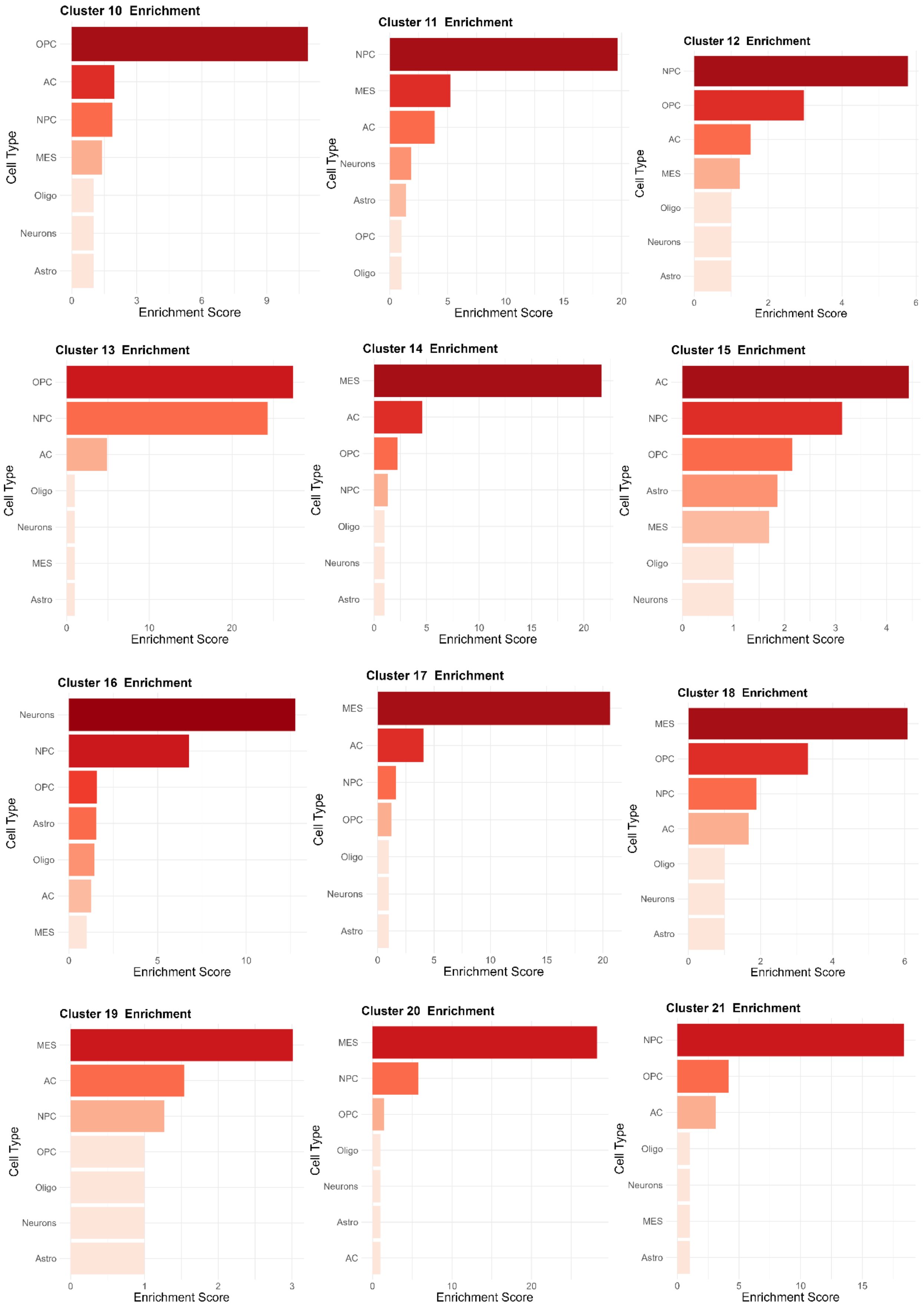
Cluster 10 Enrichment
OPC
AC
NPC
MES
Oligo
Neurons
Astro
Cell Type
0
3
6
9
Enrichment Score
Cluster 11 Enrichment
NPC
MES
AC
Neurons
Astro
OPC
Oligo
Cell Type
0
5
10
15
20
Enrichment Score
Cluster 12 Enrichment
NPC
OPC
AC
MES
Oligo
Neurons
Astro
Cell Type
0
2
4
6
Enrichment Score
Cluster 13 Enrichment
OPC
NPC
AC
Oligo
Neurons
MES
Astro
Cell Type
0
10
20
Enrichment Score
Cluster 14 Enrichment
MES
AC
OPC
NPC
Oligo
Neurons
Astro
Cell Type
0
5
10
15
20
Enrichment Score
Cluster 15 Enrichment
AC
NPC
OPC
Astro
MES
Oligo
Neurons
Cell Type
0
1
2
3
4
Enrichment Score
Cluster 16 Enrichment
Neurons
NPC
OPC
Astro
Oligo
AC
MES
Cell Type
0
5
10
Enrichment Score
Cluster 17 Enrichment
MES
AC
NPC
OPC
Oligo
Neurons
Astro
Cell Type
0
5
10
15
20
Enrichment Score
Cluster 18 Enrichment
MES
OPC
NPC
AC
Oligo
Neurons
Astro
Cell Type
0
2
4
6
Enrichment Score
Cluster 19 Enrichment
MES
AC
NPC
OPC
Oligo
Neurons
Astro
Cell Type
0
1
2
3
Enrichment Score
Cluster 20 Enrichment
MES
NPC
OPC
Oligo
Neurons
Astro
AC
Cell Type
0
10
20
Enrichment Score
Cluster 21 Enrichment
NPC
OPC
AC
Oligo
Neurons
MES
Astro
Cell Type
0
5
10
15
Enrichment Score

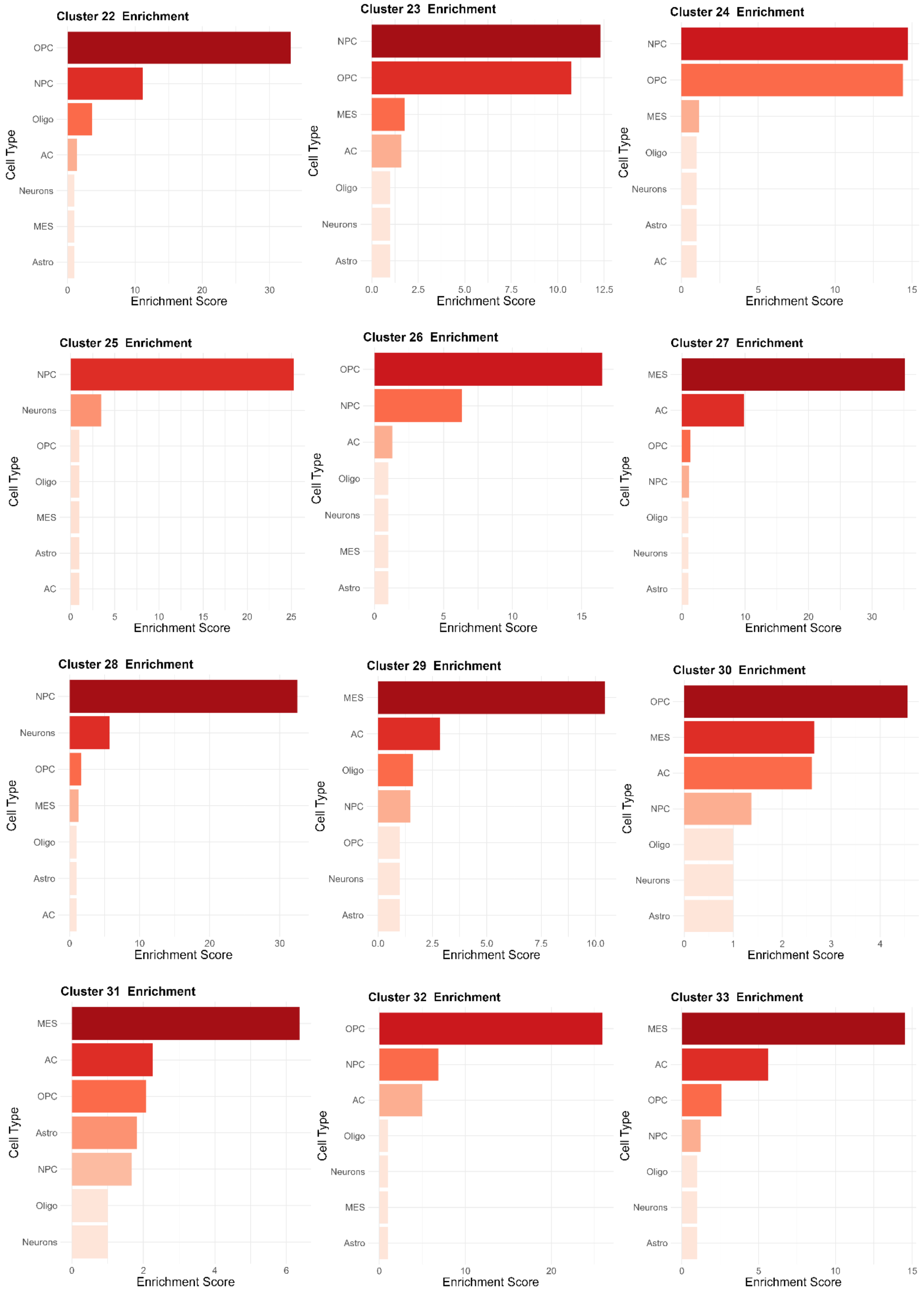
Cluster 22 Enrichment
OPC
NPC
Oligo
AC
Neurons
MES
Astro
Cell Type
0
10
20
30
Enrichment Score
Cluster 23 Enrichment
NPC
OPC
MES
AC
Oligo
Neurons
Astro
Cell Type
0.0
2.5
5.0
7.5
10.0
12.5
Enrichment Score
Cluster 24 Enrichment
NPC
OPC
MES
Oligo
Neurons
Astro
AC
Cell Type
0
5
10
15
Enrichment Score
Cluster 25 Enrichment
NPC
Neurons
OPC
Oligo
MES
Astro
AC
Cell Type
0
5
10
15
20
25
Enrichment Score
Cluster 26 Enrichment
OPC
NPC
AC
Oligo
Neurons
MES
Astro
Cell Type
0
5
10
15
Enrichment Score
Cluster 27 Enrichment
MES
AC
OPC
NPC
Oligo
Neurons
Astro
Cell Type
0
10
20
30
Enrichment Score
Cluster 28 Enrichment
NPC
Neurons
OPC
MES
Oligo
Astro
AC
Cell Type
0
10
20
30
Enrichment Score
Cluster 29 Enrichment
MES
AC
Oligo
NPC
OPC
Neurons
Astro
Cell Type
0.0
2.5
5.0
7.5
10.0
Enrichment Score
Cluster 30 Enrichment
OPC
MES
AC
NPC
Oligo
Neurons
Astro
Cell Type
0
1
2
3
4
Enrichment Score
Cluster 31 Enrichment
MES
AC
OPC
Astro
NPC
Oligo
Neurons
Cell Type
0
2
4
6
Enrichment Score
Cluster 32 Enrichment
OPC
NPC
AC
Oligo
Neurons
MES
Astro
Cell Type
0
10
20
Enrichment Score
Cluster 33 Enrichment
MES
AC
OPC
NPC
Oligo
Neurons
Astro
Cell Type
0
5
10
15
Enrichment Score

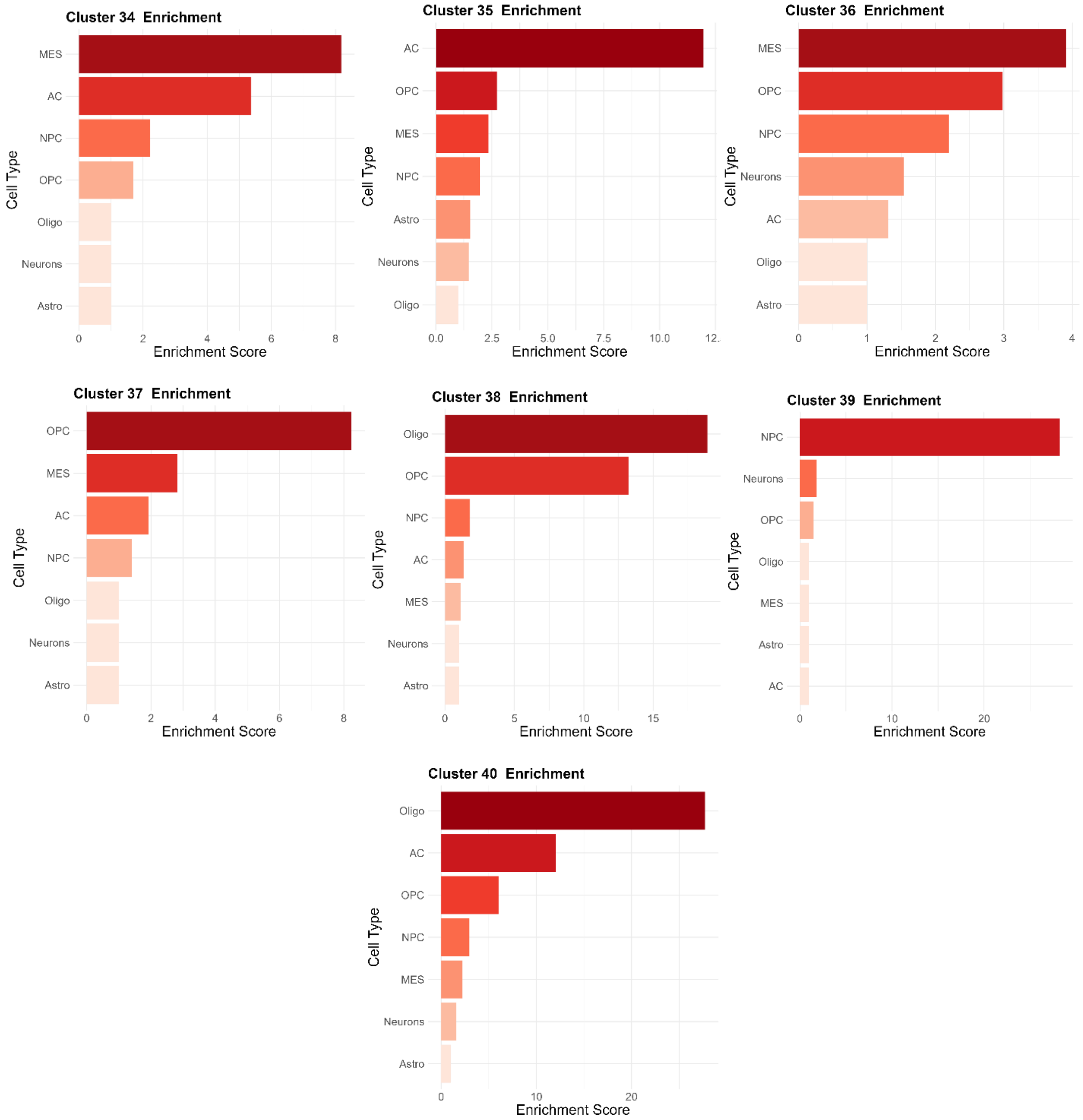

Supplementary Figure 1 (S1). Single Cell Cluster Enrichment. Clusters were labeled using meta-modules as defined by Neftel et al. (2019) [1] and Wang et al (2021) [2]. A hypergeometric test was used to evaluate whether each meta-module was overrepresented in each cluster and clusters were labeled with the most overrepresented meta-module (-log(p-value).

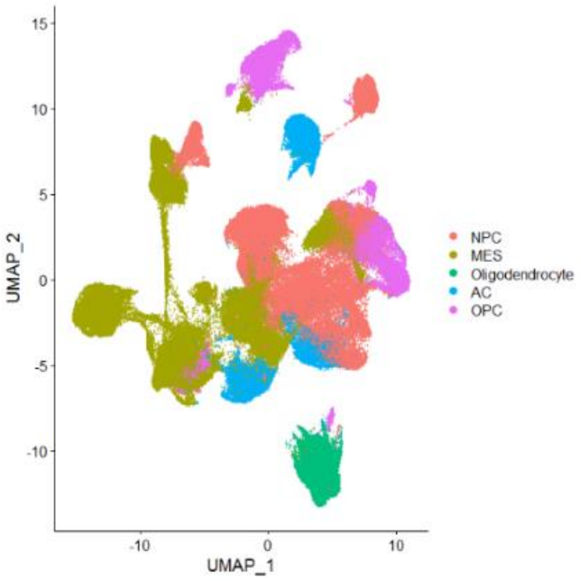

Supplementary Figure 2 (S2). UMAP of snRNA-seq Data.

Species	Logic
ABL1	= (!ATM & !CDK1 & !CDK2 & !FYN & !LCK & !PAK2 & !PRKCA & !PRKDC & !RFX1 & SRC) | (!ATM & !CDK1 & !CDK2 & !FYN & !LCK & !PAK2 & !PRKCA & !PRKDC & RFX1) | (!ATM & !CDK1 & !CDK2 & !FYN & !LCK & !PAK2 & !PRKCA & PRKDC) | (!ATM & !CDK1 & !CDK2 & !FYN & !LCK & !PAK2 & PRKCA) | (!ATM & !CDK1 & !CDK2 & !FYN & !LCK & PAK2) | (!ATM & !CDK1 & !CDK2 & !FYN & LCK) | (!ATM & !CDK1 & !CDK2 & FYN) | (!ATM & !CDK1 & CDK2) | (!ATM & CDK1) | (ATM)
ATR	ABL1
EGFR	(ABL1 | AR | EGF | FGF2 | FYN | HGF | IRF1 | JUN | JUNB | NFKB1 | RARA | RELA)
AGT	(!FOS & HIF1A) | (FOS)
AHR	AHR
AKT1	(AR | ATM | AURKA | FGF2 | LCK | PRKDC | RAC1 | SRC | TBK1 | TNF) & (!TP53 | !PPP2CA)
AKT2	(SRC | INSR | PRKDC | TBK1) & (!PPP2CA | !PTEN)
AKT3	(!MAP3K7 & !PPP2CA & !PRKCZ & !PRKDC & !PTEN & !PTK2B & !SRC & TBK1) | (!MAP3K7 & !PPP2CA & !PRKCZ & !PRKDC & !PTEN

	& !PTK2B & SRC) | (!MAP3K7 & !PPP2CA & !PRKCZ & !PRKDC & !PTEN & PTK2B) | (!MAP3K7 & !PPP2CA & !PRKCZ & !PRKDC & PTEN) | (!MAP3K7 & !PPP2CA & !PRKCZ & PRKDC) | (!MAP3K7 & !PPP2CA & PRKCZ) | (MAP3K7 & !PPP2CA)
AR	AR
ARAF	PAK1
ARNT	ARNT
ATM	(!ATR & !CDK5 & !E2F1 & !EGF & !FOXO3 & !PPP2CA & !RPS6KA2 & TNF) | (!ATR & !CDK5 & !E2F1 & !EGF & !FOXO3 & !PPP2CA & RPS6KA2) | (!ATR & !CDK5 & !E2F1 & !EGF & FOXO3 & !PPP2CA) | (!ATR & !CDK5 & !E2F1 & EGF & !PPP2CA) | (!ATR & !CDK5 & E2F1 & !PPP2CA) | (!ATR & CDK5 & !PPP2CA) | (ATR & !PPP2CA)
AURKA	(!E2F1 & PRKACA) | (E2F1)
BCL6	BCL6
BDNF	TNF
BRAF	(!AKT1 & !AKT2 & !AKT3 & !PAK4 & PPP2CA) | (!AKT1 & !AKT2 & !AKT3 & PAK4)
BUB1	CDK1
CAMK2B	CAMKK1
CAMKK1	(!CDK6 & CDK5) | (CDK6)
CDK1	(E2F1 & E2F4) & (FOS | SP1 |!TP53 | !GSK3B | !PIN1 | !MAPK1)
CDK2	(!AKT1 & !AKT3 & !MAPK3 & MITF & !TGFB1) | (!AKT1 & !AKT3 & MAPK3 & !TGFB1) | (!AKT1 & AKT3 & !TGFB1) | (AKT1 & !TGFB1)
CDK4	(!FOS & !GSK3B & !JUN & !MAX & !MYC & !NFKB1 & !RELA & STAT3 & !YES1) | (!FOS & !GSK3B & !JUN & !MAX & !MYC & !NFKB1 & RELA & !YES1) | (!FOS & !GSK3B & !JUN & !MAX & !MYC & NFKB1 & !YES1) | (!FOS & !GSK3B & !JUN & !MAX & MYC & !YES1) | (!FOS & !GSK3B & !JUN & MAX & !YES1) | (!FOS & !GSK3B & JUN & !YES1) | (FOS & !GSK3B & !YES1
CDK6	(!GSK3B & !MAX & !MYC & RELA & !SP1) | (!GSK3B & !MAX & MYC & !SP1) | (!GSK3B & MAX & !SP1)
CDK5	FYN
CDK9	AKT1 & MAPK3
CEBPA	CEBPA

CHEK1	(AKT1 | ATM | ATR | CDK1 | E2F1 | CDK2 | MAP3K8) & (!BCL6 | !PPP2CA | !TP53)
CHEK2	CDK9 & PPP2CA
CHUK	(!AKT1 & !AKT2 & !AKT3 & !MAPK3 & !NR2C2 & !PRKCB & !PRKCE & SRC & !TP53) | (!AKT1 & !AKT2 & !AKT3 & !MAPK3 & !NR2C2 & !PRKCB & PRKCE & !TP53) | (!AKT1 & !AKT2 & !AKT3 & !MAPK3 & !NR2C2 & PRKCB & !TP53) | (!AKT1 & !AKT2 & !AKT3 & !MAPK3 & NR2C2 & !TP53) | (!AKT1 & !AKT2 & !AKT3 & MAPK3 & !TP53) | (!AKT1 & !AKT2 & AKT3 & !TP53) | (!AKT1 & AKT2 & !TP53) | (AKT1 & !TP53)
CREB1	!ATM
CREBBP	(!NFKB1 & !RUNX2 & !SMAD3 & SMAD4 & !YY1) | (!NFKB1 & !RUNX2 & SMAD3 & !YY1) | (!NFKB1 & RUNX2 & !YY1) | (NFKB1 & !YY1)
CSK	(!CREBBP & !PRKAR2A & !PRKAR2B & VEGFA) | (!CREBBP & !PRKAR2A & PRKAR2B) | (!CREBBP & PRKAR2A) | (CREBBP)
CSNK1E	PLK1
CSKN1A1	(!PPP3CA & PPP3CB) | (PPP3CA)
CSNK2A1	(MAPK14 & !NOLC1)
DAPK1	!RPS6KA3
DDX58	!CSNK2A1
DYRK2	ATM
E2F1	ATM
EGF	(!FYN & LCK) | (FYN)
E2F4	E2F4
EGR1	EGR1
ELK1	ELK1
ETV1	ETV1
FGF2	(HIF1A | STAT1 | TNF) & (!TP53 | !SRC)
FOS	FOS
FOXO1	FOXO1
FOXO3	FOXO3
FYN	CSK
GRK5	CDK1
GSK3A	PRKCZ
GSK3B	(!AKT1 & !AKT2 & !AKT3 & MAPK1 & !PRKACA & !PRKCA & !PRKCZ & !RPS6KA5)
HGF	OSM & !PTEN
IGF1R	!TP53
HIF1A	ATM

IKBKB	(!AKT1 & !AKT2 & !AKT3 & !NR2C2 & !PLK1 & !PRKCA & !PRKCB & !PRKCE & !PRKCZ & !SRC & TBK1) | (!AKT1 & !AKT2 & !AKT3 & !NR2C2 & !PLK1 & !PRKCA & !PRKCB & !PRKCE & !PRKCZ & SRC) | (!AKT1 & !AKT2 & !AKT3 & !NR2C2 & !PLK1 & !PRKCA & !PRKCB & !PRKCE & PRKCZ) | (!AKT1 & !AKT2 & !AKT3 & !NR2C2 & !PLK1 & !PRKCA & !PRKCB & PRKCE) | (!AKT1 & !AKT2 & !AKT3 & !NR2C2 & !PLK1 & !PRKCA & PRKCB) | (!AKT1 & !AKT2 & !AKT3 & !NR2C2 & !PLK1 & PRKCA) | (!AKT1 & !AKT2 & !AKT3 & NR2C2 & !PLK1) | (!AKT1 & !AKT2 & AKT3 & !PLK1) | (!AKT1 & AKT2 & !PLK1) | (AKT1 & !PLK1)
IL1B	(!FOS & !JUN & !NFKB1 & !RELA & !STAT1 & STAT3) | (!FOS & !JUN & !NFKB1 & !RELA & STAT1) | (!FOS & !JUN & !NFKB1 & RELA) | (!FOS & !JUN & NFKB1) | (!FOS & JUN) | (FOS)
INSR	(!CEBPA & PRKCE) | (CEBPA)
IRF1	IRF1
JUNB	JUNB
JUN	JUN
LCK	FYN & MAPK3) | (!CSK)
MAFB	MAFB
MAP2K1	(EGFR | MAP3K7 | PAK2 | RAC1) & (!CDK1 | !PPP2CA)
MAP3K7	(!PPP2CA) | (TRAF6)
MAP3K8	AKT1
MAPK1	(FGF2 | YES1 | IL1B | PRKCA | !SGK1) & (!HGF)
MAPK10	!CDK5 & TRAF6
MAPK12	(LCK & EGF & CDK5) | (PDPK1 & PGR)
MAPK11	PAK1 & TRAF6
MAPK13	PGR
MAPK14	MAP3K7 & MAPK11 & TRAF6
MAPK8	(PRKAA1 | PRKAA2 | PRKDC | PRKCZ) & (MAP2K1 & MAP3K7) & (HGF | TNF | BDNF) & (RAC1 | TGFBR2 | PTK2B)
MAPK3	(MAPK1 & (AHR | FGF2 | ETV1 | MAFB) & (!WT1))
MAPKAPK2	(!MAPK12 & TNF) | (MAPK12)
MAX	MAX
MITF	MITF
MYC	MYC
NFATC1	NFACT1

NFKB1	NFKB1
NOLC1	RELA
NR2C2	NR2C2
OSM	STAT3
PAK1	PDPK1
PAK2	(!PRKCE & !SRC & TGFB1) | (!PRKCE & SRC) | (PRKCE)
PAK4	(!PRKCE & YES1) | (PRKCE)
PDPK1	PTEN & IGF1R
PGR	PGR
PIM1	(!PRKACA & !SP1 & !STAT1 & !STAT2 & STAT3) | (!PRKACA & !SP1 & !STAT1 & STAT2) | (!PRKACA & !SP1 & STAT1) | (!PRKACA & SP1) | (PRKACA)
PIM2	(!PRKACA & STAT3) | (PRKACA)
PIM3	STAT5A
PIN1	!DAPK1
PLK1	!ATM
PPARG	PPARG
PPP2CA	(!CREB1 & !SRC & TGFBR2) | (CREB1 & !SRC)
PPP3CA	CAMK2B
PPP3CB	CAMK2B
PRKAA1	(!CAMK2B & !CAMKK1 & MAP3K7) | (!CAMK2B & CAMKK1) | (CAMK2B)
PRKAA2	MAP3K7
PRKACA	(!AKT3 & !PPP2CA & RPS6KA5) | (AKT3 & !PPP2CA)
PRKAR2A	CDK1
PRKAR2B	CDK1
PRKCA	IL1B & !PPP2CA
PRKCB	LCK
PRKCE	FYN & !SP1 & !STAT1
PRKCZ	!PPP2CA
PRKD1	PDPK1
PRKDC	(!ATR & !EGFR & ATM) | (!ATR & EGFR) | (ATR)
PSEN1	(!CDK5 & !ELK1 & PRKCB) | (CDK5 & !ELK1)
PTEN	(TFAP2A | (TP53 & LCK) | (TP53 & PRKCA) | (PRKCA & LCK)) & (!FOS | !CSNK2A1)
PTK2B	(!CSK & !SRC & VEGFA) | (!CSK & SRC) | (CSK)
RAC1	(!ABL1 & !EGFR & !FYN & !HGF & PTK2B & !TP53) | (!ABL1 & !EGFR & !FYN & HGF & !TP53) | (!ABL1 & !EGFR & FYN & !TP53) | (!ABL1 & EGFR & !TP53) | (ABL1 & !TP53)
RARA	RARA

RFX1	RFX1
RELA	RELA
RPS6KA2	(!MAPK3 & PIM2 & RPS6KA4) | (MAPK3 & RPS6KA4)
RPS6KA3	(!FOXO1 & FYN) | (FOXO1);
RPS6KA4	(!MAPK12 & MAPK3) | (MAPK12)
RPS6KA5	(!MAP2K1 & MAPK1) | (MAP2K1)
RUNX2	RUNX2
SGK1	SRC
SMAD3	SMAD3
SMAD4	SMAD4
SP1	!ATM
SRC	(!CAMK2B & !CDK5 & !CSK & !EGF & !PRKCB & !PRKCE & VEGFA) | (!CAMK2B & !CDK5 & !CSK & !EGF & !PRKCB & PRKCE) | (!CAMK2B & !CDK5 & !CSK & !EGF & PRKCB) | (!CAMK2B & !CDK5 & !CSK & EGF) | (!CAMK2B & !CDK5 & CSK) | (!CAMK2B & CDK5) | (CAMK2B)
STAT1	STAT1
STAT2	STAT2
STAT3	STAT3
STAT5A	MAPK8
TBK1	DDX58
TFAP2A	TFAP2A
TFRC	(!ARNT & !HIF1A & MAX) | (!ARNT & HIF1A) | (ARNT)
TGFB1	(!EGR1 & !FOS & !HIF1A & !JUN & !NFKB1 & !RELA & USF1 & !YY1) | (!EGR1 & !FOS & !HIF1A & !JUN & !NFKB1 & RELA & !YY1) | (!EGR1 & !FOS & !HIF1A & !JUN & NFKB1 & !YY1) | (!EGR1 & !FOS & !HIF1A & JUN & !YY1) | (!EGR1 & !FOS & HIF1A & !YY1) | (!EGR1 & FOS & !YY1) | (EGR1 & !YY1)
TGFBR2	PDPK1
TNF	(!CREBBP & !FOS & !JUN & !NFATC1 & !NFKB1 & !PTEN & RELA & !SP1) | (!CREBBP & !FOS & !JUN & !NFATC1 & NFKB1 & !PTEN & !SP1) | (!CREBBP & !FOS & !JUN & NFATC1 & !PTEN & !SP1) | (!CREBBP & !FOS & JUN & !PTEN & !SP1) | (!CREBBP & FOS & !PTEN & !SP1) | (CREBBP & !PTEN & !SP1)
TP53	TP53
TRAF6	!PSEN1

USF1	USF1
VEGFA	(AR | EGR1 | ESR1 | FOS | HIF1A | JUN | MYC | NFKB1 | RELA | SMAD3 | SP1 | STAT1 | STAT3 | TFAP2A)
WT1	WT1
YES1	!CSK
YY1	YY1

Supplementary Figure 3 (S3). Boolean Logic Rules. Logical rules that govern the Boolean Model. Symbol Meanings: **&** (AND Gate), **|** (OR Gate), **!** (NOT).

a)

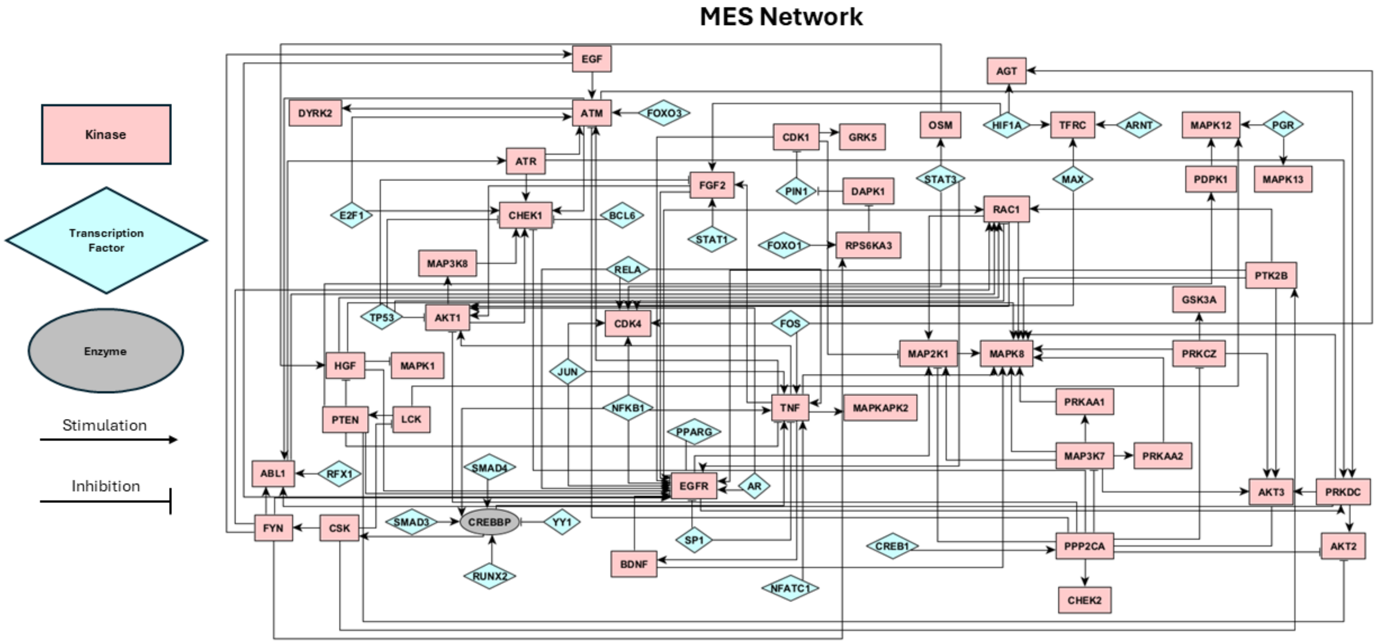

b)

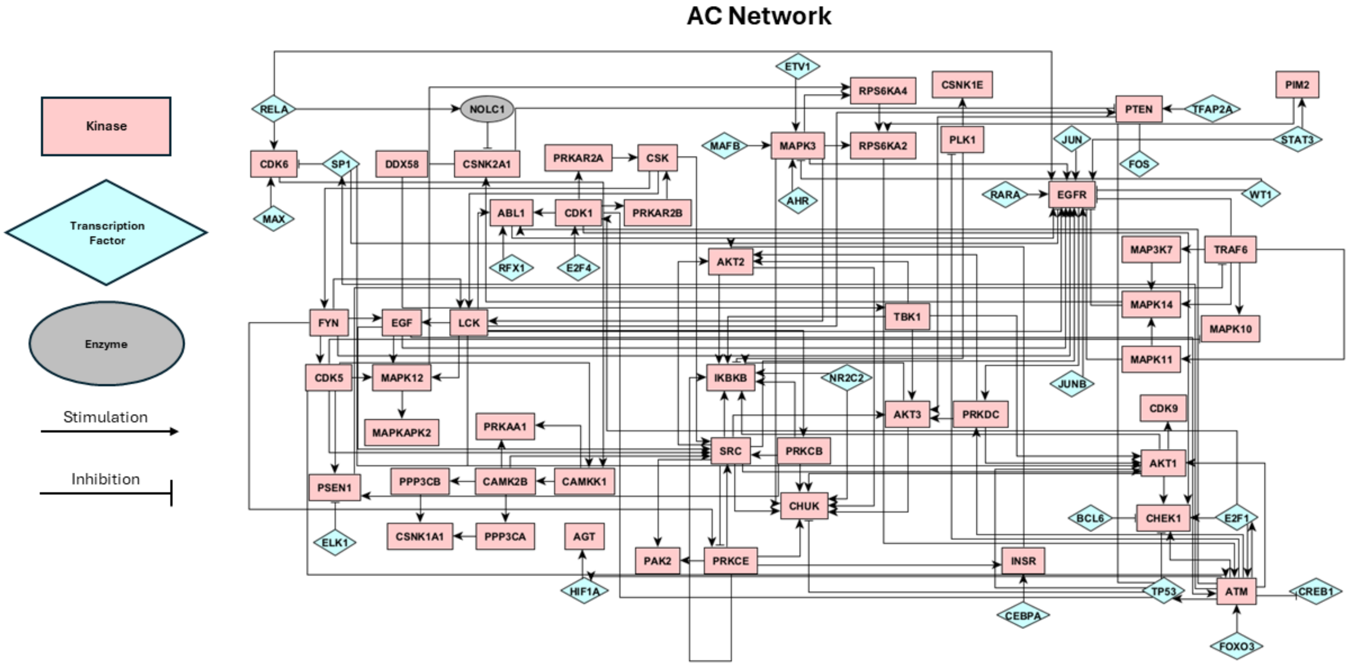
AC Network
Kinase
Transcription Factor
Enzyme
Stimulation
Inhibition
ETV1
RPS6KA4
CSNK1E
PIM2
RELA
NOLC1
PTEN
TFAP2A
MAFB
MAPK3
RPS6KA2
PLK1
JUN
STAT3
CDK6
SP1
DDX58
CSNK2A1
PRKAR2A
CSK
FOS
RARA
EGFR
WT1
MAX
AHR
ABL1
CDK1
PRKAR2B
RFX1
E2F4
AKT2
MAP3K7
TRAF6
TBK1
MAPK14
FYN
EGF
LCK
MAPK10
MAPK11
CDK5
MAPK12
IKBKB
NR2C2
JUNB
AKT3
PRKDC
CDK9
MAPKAPK2
PRKAA1
SRC
PRKCB
AKT1
PSEN1
PPP3CB
CAMK2B
CAMKK1
CHUK
BCL6
CHEK1
E2F1
ELK1
CSNK1A1
PPP3CA
AGT
PAK2
PRKCE
INSR
HIF1A
TP53
ATM
CREB1
CEBPA
FOXO3

c)

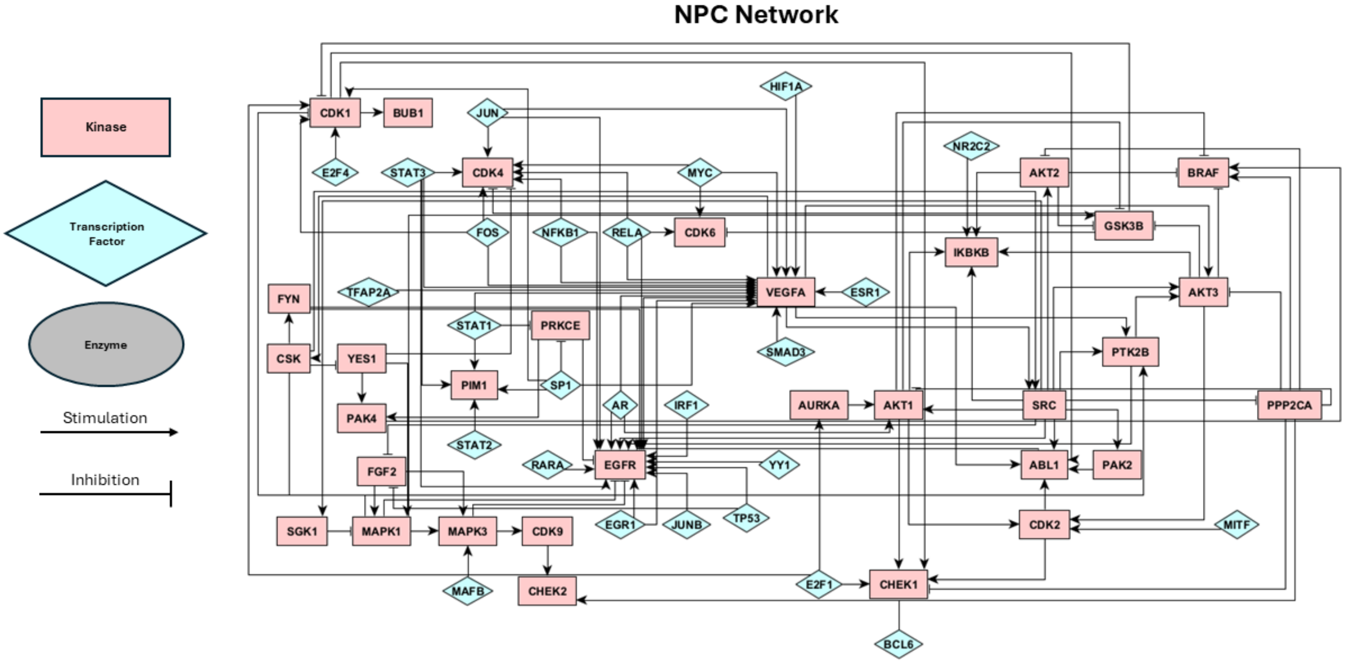
NPC Network
Kinase
Transcription Factor
Enzyme
Stimulation
Inhibition
HIF1A
CDK1
BUB1
JUN
NR2C2
E2F4
STAT3
CDK4
MYC
AKT2
BRAF
FOS
NFKB1
RELA
CDK6
GSK3B
IKBKB
FYN
TFAP2A
VEGFA
ESR1
AKT3
STAT1
PRKCE
CSK
YES1
SMAD3
PTK2B
PIM1
SP1
PAK4
AR
IRF1
AURKA
AKT1
SRC
PPP2CA
STAT2
FGF2
RARA
EGFR
YY1
ABL1
PAK2
EGR1
JUNB
TP53
SGK1
MAPK1
MAPK3
CDK9
CDK2
MITF
MAFB
CHEK2
E2F1
CHEK1
BCL6

d)

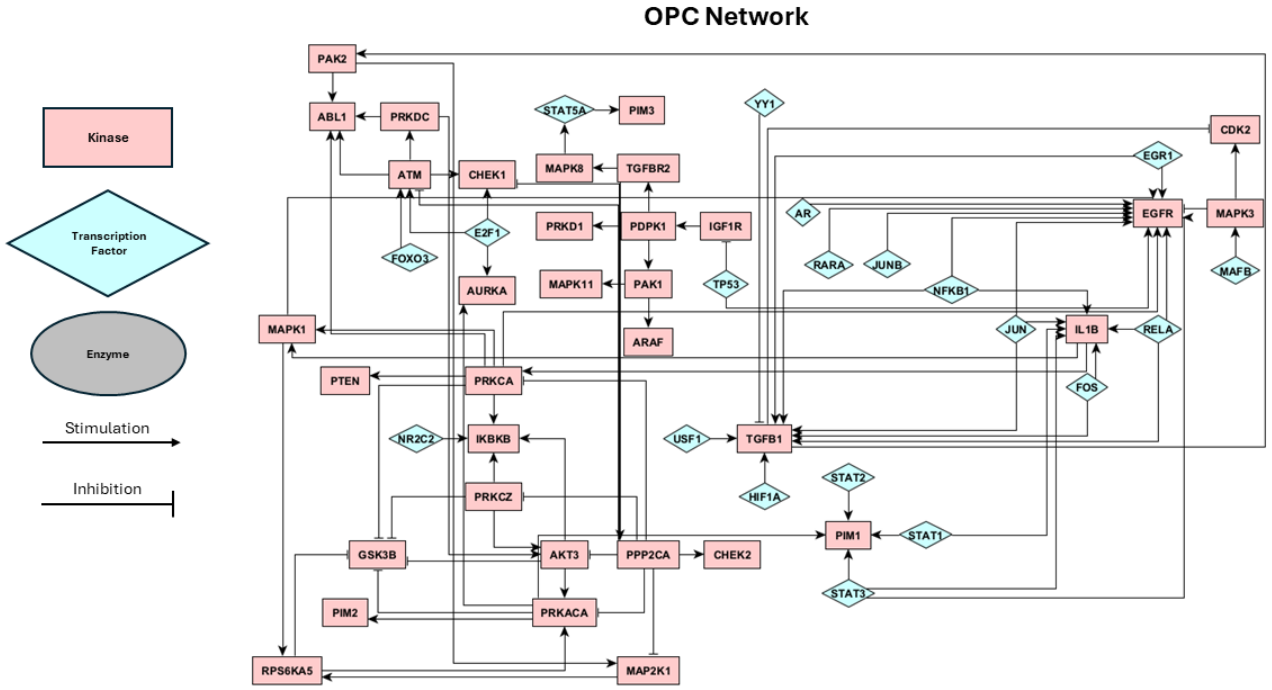

OPC Network
Kinase
Transcription Factor
Enzyme
Stimulation
Inhibition
PAK2
ABL1
PRKDC
STAT5A
PIM3
YY1
CDK2
ATM
CHEK1
MAPK8
TGFBR2
EGR1
AR
EGFR
MAPK3
PRKD1
PDPK1
IGF1R
E2F1
FOXO3
RARA
JUNB
MAFB
AURKA
MAPK11
PAK1
TP53
NFKB1
MAPK1
ARAF
JUN
IL1B
RELA
PTEN
PRKCA
FOS
NR2C2
IKBKB
USF1
TGFB1
HIF1A
STAT2
PRKCZ
GSK3B
AKT3
PPP2CA
CHEK2
PIM1
STAT1
STAT3
PIM2
PRKACA
RPS6KA5
MAP2K1

e)

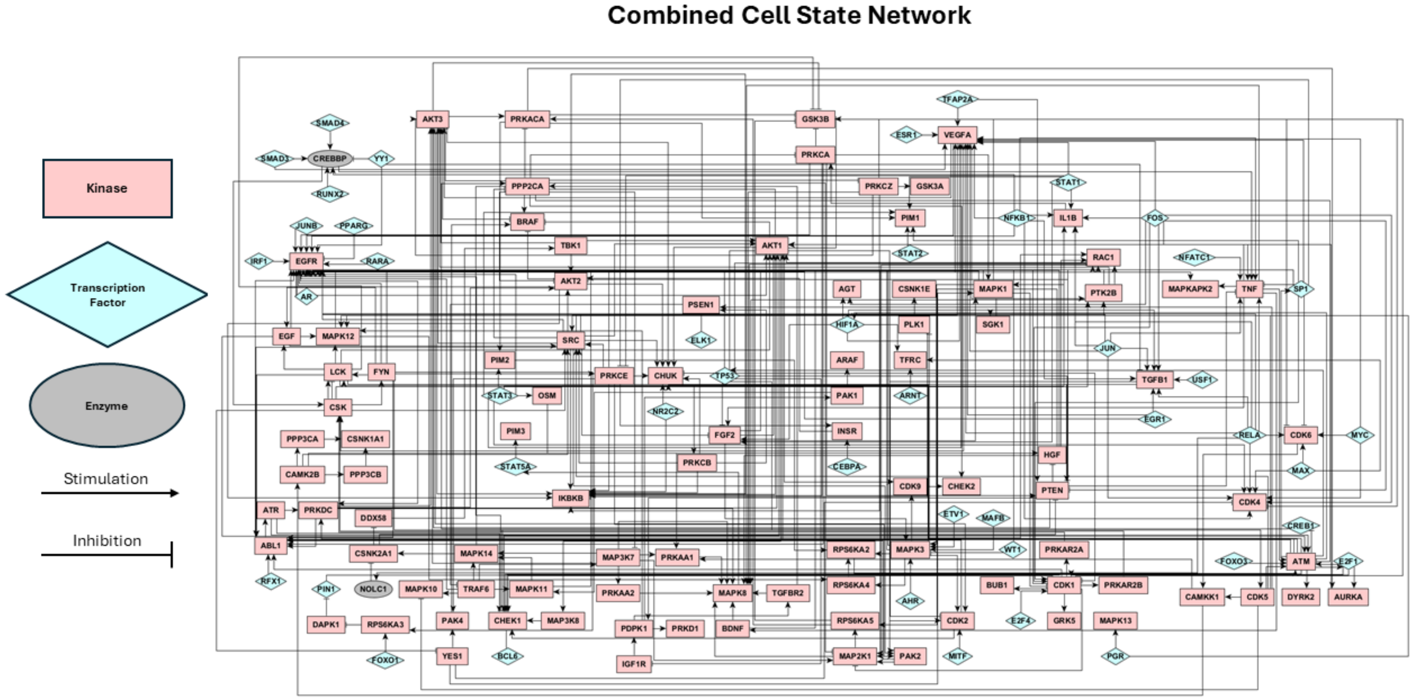

Combined Cell State Network
Kinase
Transcription Factor
Enzyme
Stimulation
Inhibition

Supplementary Figure 4 (S4). COSMOS PPINs. The PPINs of the four individual cell states (a-d) and the final combined network (e). Pink rectangles indicate kinases and blue diamonds indicate transcription factors. Activating/Stimulating interactions are depicted by arrows and inhibitory interactions are depicted by T-bars.

a) Multinomial Regression

Primary Tumors

Phenotype	Sensitivity	Specificity	Balanced Accuracy
AC	1.0<br>1.0<br>1.0	0.72<br>0.51<br>0.42	86.0%<br>75.5%<br>74.5%
MES	0.97<br>0.73<br>0.71	0.99<br>0.99<br>0.95	98.3%<br>86.4%<br>83.3%
NPC	0.41<br>0.22<br>0.14	0.99<br>0.98<br>0.98	70.2%<br>60.8%<br>56.8%
OPC	0.0<br>0.0<br>0.0	1.0<br>1.0<br>1.0	50.0%<br>50.0%<br>50.0%

Recurrent Tumors

Phenotype	Sensitivity	Specificity	Balanced Accuracy
AC	1.0<br>0.0<br>1.0	0.7<br>0.46<br>0.42	84.9%<br>23%<br>70.1%
MES	0.98<br>0.64<br>0.6	0.99<br>0.9<br>0.96	98.5%<br>77.2%<br>78.3%
NPC	0.38<br>0.14<br>0.22	0.99<br>0.99<br>0.98	68.9%<br>55.1%<br>59.1%
OPC	0.0<br>0.0<br>0.0	1.0<br>1.0<br>1.0	50.0%<br>50.0%<br>50.0%

Original Data
Data +10% Noise
Data +20% Noise

b) K-Nearest Neighbor (KNN)

Primary Tumors

Phenotype	Sensitivity	Specificity	Balanced Accuracy
AC	1.0<br>1.0<br>1.0	0.88<br>0.64<br>0.58	94.0%<br>82.0%<br>79.0%
MES	0.96<br>0.80<br>0.69	0.99<br>0.98<br>0.97	98.3%<br>88.7%<br>84.7%
NPC	0.83<br>0.43<br>0.46	0.98<br>0.98<br>0.97	90.8%<br>70.6%<br>72.0%
OPC	0.0<br>0.0<br>0.0	1.0<br>1.0<br>1.0	50.0%<br>50.0%<br>50.0%

Recurrent Tumors

Phenotype	Sensitivity	Specificity	Balanced Accuracy
AC	1.0<br>0.0<br>1.0	0.82<br>0.62<br>0.57	91.1%<br>31.0%<br>78.3%
MES	1.0<br>0.78<br>0.77	0.99<br>0.86<br>0.98	99.5%<br>82.0%<br>87.4%
NPC	0.76<br>0.32<br>0.35	0.99<br>0.99<br>0.99	87.8%<br>64.6%<br>66.6%
OPC	0.0<br>0.0<br>0.0	1.0<br>1.0<br>1.0	50.0%<br>50.0%<br>50.0%

Original Data
Data +10% Noise
Data +20% Noise

c) **Random Forest (RF)**

Primary Tumors

Phenotype	Sensitivity	Specificity	Balanced Accuracy
AC	0.0<br>0.0<br>0.33	0.99<br>0.97<br>0.96	49.5%<br>48.5%<br>64.7%
MES	0.98<br>0.98<br>0.93	0.93<br>0.91<br>0.89	95.5%<br>94.4%<br>90.9%
NPC	0.98<br>0.89<br>0.76	0.95<br>0.97<br>0.92	96.7%<br>93.1%<br>84.0%
OPC	0.0<br>0.25<br>0.25	1.0<br>0.99<br>0.96	50.0%<br>62.0%<br>60.4%

Recurrent Tumors

Phenotype	Sensitivity	Specificity	Balanced Accuracy
AC	0.0<br>0.0<br>0.0	0.95<br>0.91<br>0.92	47.3%<br>45.5%<br>45.5%
MES	0.98<br>0.98<br>0.76	0.94<br>0.96<br>0.92	96.9%<br>98.0%<br>83.6%
NPC	0.98<br>0.92<br>0.78	0.99<br>0.99<br>0.97	98.5%<br>95.2%<br>87.9%
OPC	0.27<br>0.18<br>0.27	1.0<br>1.0<br>0.96	63.6%<br>59.1%<br>61.7%

Original Data
Data +10% Noise
Data +20% Noise

d) **XGBoost**

Primary Tumors

Phenotype	Sensitivity	Specificity	Balanced Accuracy
AC	0.0<br>0.67<br>0.67	0.91<br>0.86<br>0.81	45.6%<br>76.3%<br>73.8%
MES	0.98<br>0.93<br>0.81	1.0<br>0.93<br>0.91	99.1%<br>93.2%<br>86.1%
NPC	0.89<br>0.76<br>0.70	0.95<br>0.98<br>0.94	92.3%<br>87.0%<br>82.1%
OPC	0.0<br>0.0<br>0.0	1.0<br>1.0<br>1.0	50.0%<br>50.0%<br>50.0%

Recurrent Tumors

Phenotype	Sensitivity	Specificity	Balanced Accuracy
AC	0.0<br>0.0<br>0.0	0.83<br>0.76<br>0.74	41.6%<br>38.0%<br>37.1%
MES	0.98<br>0.80<br>0.75	0.99<br>0.78<br>0.92	98.2%<br>78.7%<br>83.6%
NPC	0.81<br>0.51<br>0.67	0.99<br>0.94<br>0.91	89.8%<br>72.4%<br>79.2%
OPC	0.0<br>0.0<br>0.0	1.0<br>1.0<br>1.0	50.0%<br>50.0%<br>50.0%

Original Data
Data +10% Noise
Data +20% Noise

Supplementary Figure 5 (S5). Machine Learning Results. The sensitivity, specificity, and balanced accuracy for each machine learning model. Results are divided into two tables, one for primary tumors and one for recurrent tumors. Results from the original GLASS data is shown in red, while results from the data with 10% and 20% noise are shown in light and dark blue, respectively.

ADDITIONAL SUPPLEMENTARY FILES

Additional File 1: Differentially_Expressed_Genes.xlsx. This file contains the lists of differentially expressed genes in each of the four dominant cell states of glioblastoma. Each cell state is a separate sheet in this order: MES, NPC, OPC, AC.

Additional File 2: Differentially_Expressed_Phosphoproteins.xlsx. This file contains the lists of differentially expressed phosphoproteins in each of the four dominant cell states of glioblastoma. Each cell state is a separate sheet in this order: MES, NPC, OPC, AC.

Additional File 3: COSMOS_PPI.xlsx This file contains information regarding the PPINs for each of the four dominant cell states, as well as the final integrated network (5 total). Each network has two sheets associated with it. Sheets with the suffix "ATT" describe the attributes of the nodes of the PPIN, including the protein name and activity. Sheets with the suffix "SIF" describe the interaction between nodes, which is whether the interaction is activating or inhibiting.

References

1. Neftel, C., et al., *An Integrative Model of Cellular States, Plasticity, and Genetics for Glioblastoma.* Cell, 2019. **178**(4): p. 835-849.e21.
2. Wang, L.-B., et al., *Proteogenomic and metabolomic characterization of human glioblastoma.* Cancer Cell, 2021. **39**(4): p. 509-528.e20.
